# Is individual practice in an immersive and interactive virtual reality application non-inferior to practicing with traditional equipment in learning systematic clinical observation? A randomized controlled trial

**DOI:** 10.1186/s12909-020-02030-7

**Published:** 2020-04-22

**Authors:** Helen Berg, Aslak Steinsbekk

**Affiliations:** Department of Public Health and Nursing, Norwegian University of Science and Technology, 7491 Trondheim, Norway

**Keywords:** Virtual reality, ABCDE approach, Individual self-practice, Simulation, Clinical skills, Immersive, Interactive virtual reality, ABCDE approach, Individual self-practice, Simulation, Clinical skills, Immersive, Interactive

## Abstract

**Background:**

The aim was to investigate if individual self-practice of the ABCDE approach (Airways, Breathing, Circulation, Disability, Exposure) in an immersive and interactive virtual reality (VR) application gave non-inferior learning outcome compared to using traditional equipment (TP) in first year medical and nursing students.

**Methods:**

A non-inferior parallel group randomized controlled trial. The study was linked to a regular teaching program conducted in August and September 2019. All students participated in a 15-min ABCDE introduction session, before they self-practiced the ABCDE approach for 20 min in either a fully immersive and interactive VR application using hand controllers with some haptic feedback (Individual VR) or with blood pressure gauge, ear-thermometer and oximeter (Individual TP). The primary outcome was the number of students who documented all the eight predefined observations in the ABCDE approach in the correct order in a practical test on an advanced simulator manikin with a time limit of 5 min, done immediately after the self-practice. The predefined one-sided non-inferiority limit was 13% points.

**Results:**

Of all eligible students, 84% participated in the study and randomly allocated to VR (*n* = 149) or TP (*n* = 140). The primary outcome showed non-inferiority of the VR application with 24.8% in individual VR doing all observations in correct order compared to 27.1% TP (absolute difference 2.3% points, one sided 95% CI 2.3 to 10.8). The secondary outcomes were similar between the groups, but more students in VR reported liking the way they practiced (absolute difference 46% points, 95% CI 36.5 to 56.6) and that it was a good way to learn (36.9% points, 95% CI 26.8 to 47). VR also scored high on the System Usability Scale (mean difference 6.4% points, 95% CI 2.8–10.1).

**Conclusions:**

Individual self-practicing the ABCDE approach in VR was non-inferior to individual self-practicing with traditional equipment.

## Background

Systematic clinical observation is an essential skill to ensure patient safety and recognize deterioration in the patient [1]. The Airways-, Breathing-, Circulation, Disability-, Exposure- approach (ABCDE approach) is the internationally recommended and widely used approach for this purpose [1–3]. There is a need for implementing the ABCDE approach in undergraduate medical training [4]. According to systematic reviews, the best way to learn the ABCDE approach is through simulation-based training [5–7]. They have found both high-fidelity and low-fidelity simulations with actors, advanced and simple manikin simulators and interactive e-learning courses to be effective.

However, practical aspects challenge the quantity of simulation training. This includes the demand for high density of qualified staff, that the equipment is expensive and fragile and the activity is not available for students without arrangements [8, 9]. Furthermore, medical and healthcare students have limited opportunity to practice such skills in their clinical placements, due to lack of supervision and relevant practicing situations [10]. One possible solution to increase the opportunity for more practice is the use of virtual reality (VR) which can give the students the possibility to self-practice simulation [11]. VR may also benefit to reduce the use of critical resources like lecturers, time, cost and traveling [12].

Virtual reality with the use of head mounted goggles, is an immersive three-dimensional (3D) environment were the user is completely occluded from reality. The user can interact with the virtual environment through hand controllers [13]. Research on the effect of different types of VR applications have been collected in several meta-analyses and reviews, and these show that VR have similar effect as other forms of training like lecturing, web-based programs, video films, simulation etc. [14–18].

Thus, there can be a potential for using VR to learn the ABCDE approach. However, we have not found any publications where the ABCDE approach for systematic clinical observation is practiced in a fully immersive and interactive virtual environment. We only found two experimental studies investigating the effect of serious game where the ABCDE skills were one of the outcome measures, and these found the results to be similar as other training [19, 20].

The aim of this study was to investigate if individual self-practice of the ABCDE approach in an immersive and interactive virtual reality application (the VirSam ABCDE application) gave a non-inferior learning outcome compared to individual self-practicing with traditional equipment for skill training in first year medical and nursing students.

## Method

### Study design

This was a non-inferior parallel group open randomized controlled trial. The reason for choosing a non-inferiority design was the disadvantages of VR compared to real life skill practice. The study was part of a larger trial, where it was recruited students to another RCT simultaneously.

The trial was conducted in August and September 2019. The study was approved by the Norwegian Centre for Research Data (NSD, reference number 535088).

### Participants and recruitment

The inclusion criteria were first year medical and nursing students that had started their study no later than 2 months before this study was conducted.

The recruitment was linked to teaching programs which was integrated into the curriculum of each study program. The students were informed that they should participate in a teaching session where they would be randomized to different types of practicing the ABCDE approach. The students were informed that they would be asked to participate in this study by sharing data from the teaching session. They were asked at the end of the session to keep the focus on having the students participating in the teaching session (and not the study). Those who attended the session was eligible for the study and those who shared their data were included.

### Randomization and allocation

To randomize students, separate randomization lists were made for each batch using the RAND() function in Excel. The lists were printed as identity-stickers with an ID number and the type of practice the students were to participate in. These were sealed in identical opaque plastic bags which were mixed and randomly selected. The stickers were placed on the desk in ascending order according to the ID number. The allocation was done by asking each student entering the classroom to sequentially seat themselves at the lowest available ID number. When the introduction part was over, the students were informed where to go for their practice according to the allocation code on their sticker.

### Interventions

The whole teaching session took approximately one hour for each student; 15-min introduction, 20-min individual practice and approximately 15-min testing. The rest was time for moving between rooms or waiting for the practical test. The time for introduction and practicing is the standard of the time used in a brief and simulation phase [21, 22] in simulation training.

The focus throughout the teaching session was the importance of keeping to the ABCDE order, the eight observations to be done (Table 1) and documentation of the observations. The content used, the observations chosen and equipment provided (a digital blood pressure gauge, a digital oximeter, a digital ear thermometer, a clock, an overview of the ABCDE observations) was based on recommendations in guidelines and studies [1, 2] and in dialogue with those responsible for the curriculum at the study programs.

**Table 1:** The information the students got regarding which eight observation to do and the order they should be done in

ABCDE algorithm	Observations
A- airways	1: observe if the airways are free -document
B- breathing	2: count the respiration frequency (The number breaths per minute, one breath = inbreath + outbreath) -document
	3: get the oxygen saturation using a digital oximeter -document
C- circulation	4: get the blood pressure using a digital blood pressure gauge -document
	5: count pulse (the number of heart beats per minute) -document
D- disability	6: observe if the patient is conscious -document
E- exposure	7: get the temperature using a digital ear thermometer -document
	8: observe if the skin is normal -document

All participants took part in the introduction session where they were informed briefly about the teaching and the study. They had a six minutes lecture on the ABCDE approach and watched a seven minutes video made by the authors, demonstrating how to do the ABCDE examination on an advanced simulator manikin (The introduction video [23]).

The participants allocated to individual self-practice in VR was instructed how to take on the VR equipment (Oculus Rift S or Oculus Quest head mounted device and hand controllers) by one instructor helping 3 students. The instructor did not provide any further help except if there were technical problems with the VR equipment or the software. The VR application (the VirSam ABCDE application) was developed specifically for this study by the authors, with hired help for programming (Unity 2018.3.0f2) (Table 2, and video of the VR-features [24]). The application has a tutorial on how to use the VR hand-controllers, and an ABCDE practicing part (Table 1). In the practicing part, all observations are done on a virtual patient using virtual versions of the provided equipment. Instructions on how to do the observations is given as a silent subtitled film. Feedback on performance is automatically generated after completion of all observations.

**Table 2:** Features in the practice part of the VR application (the VirSam ABCDE application)

VR-features	Explanation
Immersion	Be presented in a 3D virtual room modelled from an equipped observation room and having 360-degree vision.
Interaction	Virtual hands to pick up and move things and to get haptic response.
Virtual patient (VP)	A healthy older male person lying on the bed half dressed, having visual response (eye blinking, head movement, open and close mouth, chest movement), haptic response (breath, pulse on the wrist), and changing clinical value responses to use of digital equipment (BP, temperature, O2 saturation). No vocal response.
Haptic feedback	Vibration in the hand controllers when feeling the pulse (each heart beat) on the wrist, and when placing the hand on the chest (each respiratory intake).
Audio feedback	Inflation sounds from blood pressure gauge and “bip” from ear thermometer when measures ready (5 s)
Wristwatch	On left hand. Classic design showing real-time including seconds.
Patient monitor	Monitor with touch screen buttons to get clinical values (BP, temperature, O2 saturation).
Documentation tablet	Tablet with touch screen buttons for responses, including numeric pad for entering clinical values and choice between predefined options.
Instructions	A silent subtitle video running on a wall mounted screen showing how to do the observations, and a poster on the wall with the ABCDE observations.
Feedback	When the user select that all documentations are done, a scoreboard appears with detailed feedback and a summary maximum of three stars, covering order of observations, whether all observations were done and if the values from the observations were correct.

The participants allocated to individual self-practice with traditional equipment was instructed what to do and how to use the equipment by one instructor helping 3 to 6 students. They got a printed sheet with pictures of the equipment along with simple instructions for its technical use. The instructors did not provide any further help except if there were failure with the equipment.

The minimum of help in both the groups was to reflect a self-training situation.

### Data collection

The participants completed a baseline questionnaire when they entered the introduction session.

The outcome data was collected through a questionnaire and a practical test after the practicing part. First, they answered a questionnaire on the right order of the ABCDE and the eight observations, and on their experiences with the different parts of the whole teaching session. There was no time limit, but the majority used approximately 6 min. Afterwards they did a full ABCDE examination on an advanced simulator (the 3G or ALS simulator, Laerdal Inc., Stavanger, Norway) with clinical values of a healthy person. The same equipment as described above was available at the bedside table. The participants were informed that they got maximum 5 min to perform the examination, at which point they were interrupted. One staff member was present to give instruction and assist with technical issues like operating the monitor to show the values from blood pressure, saturation and temperature if the students did these observations. If the staff observed that the students struggled with the technical issues without requesting help, they helped. Otherwise they did not interact with the student, they were instructed to only answer “do as you think is best” if the student asked anything. The staff was blinded to the student’s allocation.

To validate the scoring of the participants documentation of their ABCDE observations from the practical test, the authors (HB and AS) independently scored sub-samples in an iterative process. These scorings and the criteria for scoring was refined until consensus. Then HB and AS independently coded the answers from 30 randomly selected students and subsequently found some data punching errors and a few incompatible answers. It was therefore decided to enter all data twice by two independent persons. HB and a third person hired for the purpose scored all the answers independently and checked for accordance. They were blinded to the allocation in this process.

### Implementation of the intervention

To monitor the implementation of the intervention, the technical problems encountered during the self-practice was recorded. The students were also asked how many times they completed full ABCDE examinations during the practice session (0, 1, 2, 3 times or more).

### Outcome measures

The primary outcome was the number of students who documented all eight ABCDE observations in the correct order on the practical test (yes/no). When there were two observations required for one step in the algorithm (B, C, E), it was scored as correct regardless of the order the observations were documented.

One group of secondary outcomes concerned the ABCDE approach:

- Number of participants who arranged the eight observations in the right order. The order in the questionnaire was in the same random order for all students [1–8]. This was coded equal to the primary outcome.
- Number of participants who arranged the ABCDE letters, presented with their Norwegian names which does not correspond to the same letters, in the right order (yes/no). The order in the questionnaire was in the same random order for all students (DBAEC).
- Number of participants who had all eight observations documented, but ABCDE in the wrong order in the practical test.
- Number of participants who did not complete all eight observations in the practical test.
- Number of participants who did not complete all eight observations but had the right ABCDE order on the documented observations in the practical test.
- Number of participants who wrote both the type of observation and the correct type of result on all the documented observations in the practical test.
- Number of participants who wrote both the type of observation and the correct type of result on the each of the eight individual observations in the practical test.
- The average number of observations documented in the practical test.
- The average number of observations documented in the correct order counted from A (Airways) in the practical test.

The other group of secondary outcomes concerned the student’s experiences with the teaching session and where asked on the questionnaire. The questions were scored on a Likert Scale where 1 was very strong disagreement and 5 was very strong agreement. The scale was dichotomized, with 4 and 5 coded as agreement:

- Did you get enough training on the ABCDE approach before starting to practice?
- Did the video help you learn what observations to do?
- Did you have enough time to practice?
- Did you like this way to practice?
- Do you think this training and practice was a good way to learn the ABCDE approach?
- Do you feel confident to conduct an ABCDE examination?

They also completed the System Usability Scale (SUS) as a measure of the usability of the system they used during the self-practice. The answers to the ten questions were transformed into one single score according Brooke [25] and given a grade from the curved grading scale (CGS) [26].

### Statistics

Data for the sample size calculation came from previous studies testing clinical learning outcomes, indicating that a 10–15% points non-inferiority limit is fair [27, 28], and it was decided on a limit of 13% points. We conducted a pilot with 18 health worker students in their second year at a vocational high school who had some experience in systematic clinical observation. Twenty percent of these students got everything correct on the primary outcome. We expected a similar outcome arguing that the university students in our study had less practical experience, but more experience in studying to master new tasks. With an expected outcome in both groups of 20% correct answers, with a non-inferiority limit of 13%, a power (1-B) of 80% and significance level (alpha) of 0.05, 118 students was needed in each group. The calculation was done using the web calculator for non-inferior trials provided by Sealed Envelope [29, 30].

Baseline variables are presented with descriptive statistics. As there was no deviation from the allocated groups and hardly any missing data, only analysis of available data was done. It was used independent sample t-tests for continuous variables (SPSS v. 26, IBM Inc) and tests of proportions using two-sample test of proportions for the categorical variables (StataMP 16, Stata Inc). Results are presented as an absolute difference. For the primary outcome the one-sided 95% confidence interval (95% CI) is reported according to the one-sided non-inferiority limit. To make the presentation more conventional, the secondary outcomes are reported with two-sided 95% CI.

## Results

### Recruitment and baseline characteristics

**Fig. 1:**
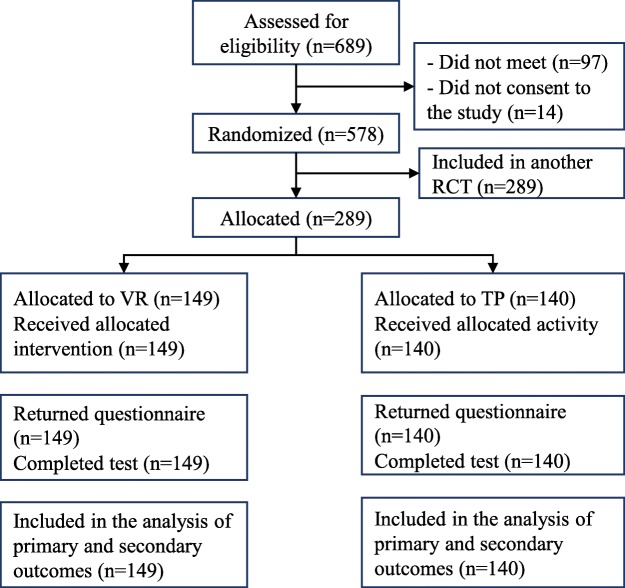
Flow of participants (VR; virtual reality, TP; traditional practice)

Overall 689 first year medical and nursing students were eligible in the larger study and 289 participated in this study (another 289 participated in another RCT). They were randomized with 149 to individual self-practice in virtual reality (Individual VR) and with 140 to individual self-practice with traditional equipment (Individual TP) (Fig. 1).

There were 227 (78.5%) females and the majority was from 20 to 24 years old (Table 3). A total of 243 (87.7%) of the participants reported to have been taught cardiopulmonary resuscitation (CPR) previously. There were 33 (11.9%) participants reported to have conducted a systematic clinical observation before, and 64 (23.1%) of the participants reported to have been taught the ABCDE approach previously. Some had experience in using VR (72 (26%)) or simulator manikin (112 (40.4%)).

**Table 3:** Baseline characteristics of the participants. The *n* for each variable can vary due to missing, *n* (%)

Baseline variables	All (***N*** = 289)	VR group (***N*** = 140)	TP group (***N*** = 149)
Gender			
-Male	50 (17.3)	28 (20.3)	22 (15.8)
-Female	227 (78.5)	110 (79.7)	117 (84.2)
Age			
-Under 20 year	66 (23.8)	25 (18.1)	41 (29.5)
-20–24 year	179 (64.6)	96 (69.6)	83 (59.7)
-Over 25 year	32 (11.6)	17 (12.3)	15 (10.8)
Study program			
-Medicine	69 (23.9)	36 (24.2)	33 (23.6)
-Nursing	220 (76.1)	113 (75.8)	107 (76.4)
Have you previously (number answering yes):			
-Worked in health care	157 (56.7)	80 (58.0)	77 (55.4)
-Been taught cardiopulmonary resuscitation (CPR)	243 (87.7)	122 (88.4)	121 (87.1)
-Conducted systematic clinical observation	33 (11.9)	18 (13.0)	15 (10.8)
-Been taught the ABCDE-approach	64 (23.1)	38 (27.5)	26 (18.7)
-Used a blood pressure gauge	119 (43.0)	61 (44.2)	58 (41.7)
-Counted respiration frequency on someone else	112 (40.4)	52 (37.7)	60 (43.2)
-Tried virtual reality googles	72 (26.0)	39 (28.3)	33 (23.7)
-Trained using a simulator manikin	112 (40.4)	48 (34.8)	64 (46.0)

Those in individual TP was somewhat younger (Table 3). A larger proportion in VR had been taught the ABCDE approach but fewer had used a simulator manikin. On balance, the groups were similar at baseline (Table 3).

### Implementation of intervention

The intervention in both groups was implemented as planned, without any major technical or other type of practical problems. It was only recorded that VR-goggles had to be restarted three times because of lost tracking of hand-controller.

A larger proportion of the students practicing in the VR application reported completing the full ABCDE examination two times or more during the practice session (absolute difference 30.4% points, 95% CI 21.5 to 39.4).

### Primary outcome

**Fig. 2:**
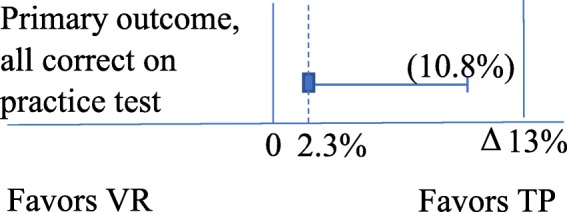
Primary outcome

A total of 37 (24.8%) participants in VR and 38 (27.1%) in TP had all eight observations documented in the correct ABCDE order. The absolute difference was 2.3% points, the one-sided 95% CI upper level was 10.8% which was within the limit of 13% points and thus demonstrate the non-inferiority of VR practice (Fig. 2).

### Secondary outcomes

Except for one outcome, the secondary outcomes concerning the ABCDE approach showed similar results which further strengthened the non-inferiority of VR practice (Table 4). The secondary outcome in the questionnaire, which was similar to the primary outcome, having all observations in the correct order, showed an absolute difference of 7.8% points (95%CI − 3.6 to 19.1) in favor of TP. For the other outcomes, it varied whether the small differences favored VR or TP. The outcome which showed a difference was the reporting of Respiratory frequency where students in VR did better (97.3% correct in VR vs 89.3% in TP, absolute difference 8% points, 95% CI 2.3 to 13.8).

**Table 4:** Secondary outcomes measures concerning the ABCDE approach. Numbers are *n* (%) or mean (SD) with difference between the groups and 95% confidence interval (95%CI)

Outcome measure	Individual VR ***N*** = 149	Individual TP ***N*** = 140	Absolute diff. % points (95% CI)	***P***-value
Number of participants that in the questionnaire had:				
- all eight observations in correct ABCDE order	81 (54.4)	87 (62.1)	7.8 (−3.6 to 19.1)	0.180
- ABCDE in the right order	136 (91.3)	127 (90.7)	0.6 (−7.2 to 6.0)	0.868
Number of participants in the practical test who:				
- had all eight observations documented, but ABCDE in wrong order	6 (4.0)	5 (3.6)	0.5 (−4.9 to 4.0)	0.840
- did not complete all eight observations	106 (71.1)	97 (69.3)	1.9 (−12.4 to 8.7)	0.730
- did not complete all eight observations, but had the right ABCDE order on the documented observations	55 (36.9)	56 (40.0)	3.1 (−8.1 to 14.3)	0.590
- wrote both the type of observation and the result of the observation	89 (59.7)	86 (61.4)	1.7 (−9.6 to 13.0)	0.768
Number of participants in practical test with correct observation of (independent of order):				
- Airways	145 (97.3)	135 (96.4)	0.9 (−4.9 to 3.1)	0.664
- Respiratory frequency	145 (97.3)	125 (89.3)	8.0 (2.3 to13.8)	0.006
- Saturation	141 (94.6)	135 (96.4)	1.8 (−3.0 to 6.5)	0.461
- Blood Pressure	144 (96.6)	137 (97.9)	1.2 (−2.5 to 5.0)	0.530
- Pulse	118 (79.2)	121 (86.4)	7.2 (−1.4 to 15.9)	0.104
- Disability	93 (62.4)	84 (60.0)	2.4 (−13.7 to 8.8)	0.674
- Temperature	84 (56.4)	82 (58.6)	2.2 (−9.2 to 13.6)	0.706
- Skin	77 (51.7)	64 (45.7)	6.0 (−17.5 to 5.5)	0.311
Average number of observations documented from practical test	Mean 6.3 SD (1.5)	Mean 6.4 SD (1.4)	Mean diff. 0.05 95%CI (−0.382 to 0.285)	0.775
Average number of observations documented in the right order from A (Airways) in practical test	Mean 5.1 SD (2.5)	Mean 5.2 SD (2.3)	Mean diff. 0.07 95%CI (− 0.623 to 0.478)	0.796

For the secondary outcomes concerning the experiences with the teaching session, there was a difference on the satisfaction with the type of practice (Table 5). Students practicing VR scored higher on liking the type of practice (82.6% vs. 36%, absolute difference 46.6% points, 95% CI 36.5 to 56.6) and on the training and practice were a good way to learn the ABCDE approach (85.1% vs. 48.2%, absolute difference 36.9% points, 95% CI 26.8 to 47.0) (Table 5). Furthermore, the outcome on the SUS favored VR with a mean SUS score of 79.7, corresponding to Grade A-, and 73.3, Grade B-, in TP (absolute difference 6.4% points, 95% CI 2.8 to 10.1).

**Table 5:** Secondary outcomes measures concerning the students experiences with the teaching session. Numbers are *n* (%) or mean (SD) with difference between the groups and 95% confidence interval (95%CI)

Outcome measure	Individual VR ***N*** = 149	Individual TP ***N*** = 140	Absolute diff. % points (95%CI)	***P***-value
Number of participants who thought:				
- they got enough training about ABCDE before starting practicing	36 (24.2)	29 (20.7)	3.4 (−13.1 to 6.2)	0.483
- they learned what observations to do trough the introduction video	83 (55.7)	81 (57.9)	2.2 (−9.3 to 13.6)	0.712
- they had enough time to practice	48 (32.7)	56 (40.3)	7.6 (−3.5 to 18.8)	0.180
- the way to practice was likable	123 (82.6)	50 (36)	46.6 (36.5 to 56.6)	< 0.001
- the training and practice were a good way to learn the ABCDE approach	126 (85.1)	67 (48.2)	36.9 (26.8 to 47.0)	< 0.001
- they were confident to conduct an ABCDE examination	43 (28.9)	37 (26.4)	2.4 (−12.7 to 7.9)	0.644
System usability scale (SUS, range 0–100)	Mean 79.7 SD (14.6)	Mean 73.7 SD (16.2)	Mean diff. 6.0 95% CI (2.8 to 10.1)	< 0.001

## Discussion

Self-practicing the ABCDE approach individually in VR using the VirSam ABCDE application gave a non-inferior learning outcome compared to individual self-practicing with traditional equipment. Most of the other outcomes gave similar results in both groups, but those practicing in VR was more satisfied and scored the usability higher.

The main strength of this study is the design and the high proportion of students participating, making the results generalizable to similar first year medical and nursing students. The study also included the required number of students from the sample size calculation. There were no major methodological limitations except for the lack of blinding due to the nature of the study. The study tested only one type of VR application, had short follow up time and an larger effect could be expected with more time to practice and/or repetition as it is known that repeated practice is the best way to retain knowledge and skills [31, 32]. Another limitation is that there was no systematical measure on environmental impact in this study, which could have been warranted given the investigation of VR which is a relative new type of equipment. On balance we do not see that the environmental impact of VR compared to traditional real-life practice (e.g. travel needed for face-to-face practice) is likely to be different, given relative low cost and the same type of resources needed for accomplishment of the interventions in this study.

The study confirmed the a priori assumption that VR would give a non-inferior learning outcome due to VR having some disadvantages compared to practicing in real life. Only a few earlier randomized controlled trials with a non-inferior assumption was found [33–35], and they got a similar result as the current study. Other randomized controlled trials without a non-inferior assumption have found the use of VR to give similar learning outcome as the comparison intervention [16, 36, 37]. However, there are some recent meta-analysis that have found VR to be superior, e.g. comparing VR simulator to box trainer for minimally invasive procedures [15] and in improving postintervention knowledge and skills [18]. Thus, the current study, due to its size and rigor, lends strong support to the assumption that VR at least gives a non-inferior learning outcome.

The students evaluated the usability of the VR application, as documented by the System Usability Scale (SUS), to be better than practicing with traditional equipment. The VR application got a SUS rating equal to a A-, which is in the 85–89 percentile [26]. This is encouraging as there are earlier studies showing that VR can give users motion sickness, that technical problems like software failure can happened and that the effort of learning to use VR can be distracting to learning new skills [20, 38]. Furthermore, the high usability scores confirm our observation that there were no technical problems. But more importantly, it is a clear indication that the features of the VirSam ABCDE application developed for this study was well chosen and implemented.

The main features of the VirSam ABCDE application are immersion and interactivity, which have been found important for creating engagement and satisfaction in VR [19, 39]. Most studies on the effect of VR evaluated in clinical- and healthcare reviews and meta-analyses have used either fully immersive [33, 34] or interactive [36] solutions. Thus, there are few studies on the effect of applications that are both immersive and interactive. This was one of the reasons that the application used in this study was self-developed, no application was found that was both fully immersive and interactive and focusing on the ABCDE approach.

Due to the non-inferiority on learning outcomes and superiority on satisfaction and usability, the application can be recommended for use in education of healthcare professionals. Nevertheless, the application has some restrictions. It was emphasized that the level of the ABCDE learning objective should be adapted to novices, and that the VR application should be easy use. Thus, it is likely not to be relevant for personnel experienced in using the ABCDE approach nor in training for the more advanced ABCDE observations.

## Conclusions

Self-practicing the ABCDE approach in VR gave a non-inferior learning outcome compared to self-practicing with traditional equipment in first year medical and nursing students, and the students were more satisfied with using VR. VR solutions that is immersive and interactive can be used as a practical and engaging way to learn the fundamentals of the ABCDE approach.

## Data Availability

The datasets analyzed during the current study are available from the corresponding author on reasonable request.

## Abbreviations

ABCDE: Airways, Breathing, Circulation, Disability, Exposure
VR: Virtual Reality
TP: Traditional Practice
SUS: System Usability Scale
CGS: Curved Grading Scale
CI: Confidence Interval
SD: Standard Deviation
3D: Three-Dimensional
CPR: Cardiopulmonary Resuscitation
